# Conference Reports: ELEVENTH NATIONAL COMPUTER SECURITY CONFERENCE Baltimore, MD October 17–20, 1988

**DOI:** 10.6028/jres.094.025

**Published:** 1989

**Authors:** Elizabeth B. Lennon

**Affiliations:** National Computer Systems Laboratory, National Institute of Standards and Technology, Gaithersburg, MD 20899

The National Computer Systems Laboratory (NCSL) and the National Computer Security Center (NCSC) of the Department of Defense co-sponsored the Eleventh National Computer Security Conference held in Baltimore, Maryland on October 17–20, 1988. More than 1600 attendees from government, industry, and academia participated in the four-day conference which provided a forum for the exchange of ideas and technology in computer security issues. The theme of the conference—”Computer Security … Into the Future”—reflected the growth of computer security awareness as well as parallel advances in research, vendor products, emerging areas of special interest, and planning for the future. NCSL’s Irene Gilbert and NCSC’s Eliot Sohmer co-chaired the program committee of the conference.

## 1. History of the Conference

Since the passage of the Brooks Act in 1965, the National Computer Systems Laboratory (formerly the Institute for Computer Sciences and Technology) has played a vital role in protecting the security and integrity of information in government computer systems through its Computer Security Program. Computer security standards and guidelines addressing the cost-effective protection of unclassified information have been issued since 1972. Working with a broad spectrum of organizations from government, industry, academia, and voluntary standards groups, NCSL advances the development of standards, develops test methods, transfers technology to potential users, and provides technical assistance to government and industry in computer security applications. The Computer Security Act of 1987 strengthened NCSL’s role in protecting sensitive information in federal government computers.

Computer security initiatives in the Department of Defense (DoD) parallel those of NCSL. In 1978, the Assistant Secretary of Defense for Communications, Command, Control and Intelligence established a Computer Security Initiative to ensure the widespread availability of trusted ADP systems within the DoD community. In 1981, the Department created the National Computer Security Center and charged the new organization with responsibility for administering the activities of the Initiative. The Center advances the development of trusted computer systems and publishes guidelines on computer security issues. In response to the increasing importance of computer security in government and industry in recent years, the DoD Computer Security Initiative has expanded to support basic research for the development of additional trusted systems. DoD’s technology transfer program ensures that other federal agencies and the private sector benefit from advances in computer security technology.

To pursue common goals and to address mutual concerns, NCSL and NCSC joined forces in 1979 to co-sponsor the first National Computer Security Conference held at the National Bureau of Standards. Dr. Dennis Branstad, NCSL (now a NIST Fellow) and Mr. Stephen Walker, then Chairman, Computer Security Technical Consortium, organized the first seminar as an information exchange on computer security issues. The Conference has grown significantly in size and scope since its inception 11 years ago. This growth parallels the increase in security awareness in information processing systems and the greater interest in the existing and emerging technology available to protect vital information in computer systems.

## 2. The Eleventh National Computer Security Conference

The 1988 Conference offered participants a variety of presentations and panel discussions providing insight on the use of today’s technology to protect vital information and on planning for tomorrow’s needs by understanding the products and services to be available in the future. The first-day “Overture” presentations and tutorials encouraged newcomers to gain an overview of selected introductory topics such as the “Orange Book” (DoD Trusted Computer System Evaluation Criteria), trusted network interpretation, Dockmaster, and risk management activities.

The second day of the conference opened with welcoming remarks from NCSL Director James H. Burrows and NCSC Director Patrick R. Gallagher. Following the keynote address by the Honorable Tom McMillan, Democratic Representative from Maryland, a plenary session on the Computer Security Act of 1987 focused on how NIST and the National Security Agency will interact on policy, technology, and implementation of the legislation. Participants in the plenary session included James Burrows, NCSL; Patrick Gallagher, NCSC; Eliot Sohmer, NCSC; Dennis Branstad, NCSL; Jerry Rainville, NSA; Stuart Katzke, NCSL; and moderator Stephen Walker, Trusted Information Systems. Among the subjects addressed in the plenary session were the implementation activities at NIST and NSA, the Memorandum of Understanding between the two agencies, use of resources, and FY89 plans; impact of the Computer Security Act on NSDD-145; trusted system technology; cryptography; INFOSEC products; trusted network interpretation/trusted database interpretation; trusted UNIX activities; and international security standards and export control.

Following this overview, speakers from the private and public sectors addressed the major computer security issues facing government and industry today and in the future. Selected presentations are summarized below.

### 2.1 Models and Modeling Integrity

Several papers described security policies and formal policy models for computer systems. T. Keefe and W. Tsai of the University of Minnesota joined with M. Thuraisingham of Honeywell to present a paper describing a security model for a multilevel secure object-oriented system. The model supports a data sensitivity level classification appropriate for use in Multilevel Secure Database Management Systems (MLS/DBMS). The advantage of this model is that it allows a subject to act with the lowest clearance level necessary to accomplish a task, avoiding the over-classification of data. G. Dinolt, J. Freeman, and R. Neely of the Ford Aerospace Corporation reported on a security policy and formal policy model for an internet system. The model provides a view of the internet system as a whole, not as a collection of components, and was developed in accordance with requirements of the DoD “Orange Book.” This research illustrates a way of modeling the security attributes of an internet system which has been used in the production of the formal specification of the Multinet Gateway System and in the formal verification of that specification.

Representatives from Odyssey Research Associates, Inc. presented a paper describing the Ulysses computer security modeling environment. Ulysses is a collection of tools that assist in the design and verification of secure computer systems. The design methodology supported by Ulysses uses the same principles of modularity and reusability that characterize modern programming development environments. Because of its overall system design, Ulysses has the potential to significantly reduce the time and effort needed in constructing secure models.

The next series of papers addressed the application of computer security technology to integrity issues. David D. Clark of M.I.T. outlined an evaluation criteria for integrity as defined by the Clark/Wilson model, which proposes an integrity policy based upon separation of duties, well-formed transactions, and audit trail to meet the needs of business and the non-defense sector. William R. Shockley, Gemini Computers, spoke on implementing the Clark/Wilson integrity policy using current technology. He described a methodology for converting a policy in Clark/Wilson notation into a corresponding mandatory policy expressed as a lattice of access classes with supporting identification and authentication policies. Shockley concluded that the Clark/Wilson integrity requirements can be met by existing, appropriately configured Trusted Computing Bases. Finally, Paul A. Pittelli of the Department of Defense presented a paper which defined a security policy for data integrity which is expressed in non-interference theory.

### 2.2 Risk Management

The risk management process enables computer users and managers to analyze information assets, threats, and vulnerabilities, to determine the measure of risk, and to select cost-effective safeguards for reducing the risks. The session on risk management was chaired by Donna Dodson of NCSL. The first speaker was Thomas W. Osgood, Manager, Security Assurance, Computer Sciences Corporation, who described a model for ADP risk analysis in the military environment typified by the Defense Communications Agency Joint Data Systems Support Center in the Pentagon. The methodology presented uses multiple metrics, a standardized threat nomenclature, and standardized reporting to meet the requirements of both a single-site, single-system environment and the more typical multiple-site, multiple-system environments of most military commands. A second paper by Martin Marietta researchers H. Mayerfeld and E. Troy described a new artificial-intelligence-based approach to standardizing and automating the risk management process, resulting in risk assessments that are more objective, uniform, and cost-effective. This approach uses a four-level abstraction hierarchy for classifying system components and assets to construct system models; risk to informational assets is determined according to the criteria of confidentiality, integrity, and availability.

Modeling security risk in networks was the subject of a talk by Howard L. Johnson, Information Intelligence Sciences, Inc., and J. Daniel Layne, Computer Technology Associates, Inc. Distributed secure systems also have distributed security policy and unequal security risk. In addressing the complex subject of risk analysis in a distributed system, Johnson and Layne’s approach expands the Orange Book concept of the primary external interface as the human “user” to include all “external subjects” such as humans, host computers, networks, other components, and other systems. This risk evaluation methodology has been programmed to simulate many different system environments.

### 2.3 Audit and Intrusion Detection

Since no combination of technologies can prevent legitimate users from abusing their authority in a computer system, auditing utilizes automated tools to analyze the vast amount of audit data for suspicious behavior by users. The next series of talks focused on the use of audit and intrusion detection in computer security. Teresa Lunt of the SRI International Computer Science Laboratory presented a survey of the automated audit trail analysis techniques and intrusion-detection systems currently available. One particularly effective approach is a statistical user profile augmented with a rule-base that characterizes intrusions. Ms. Lunt concluded that auditing and intrusion-detection mechanisms are useful in detecting the less skilled penetrator because they increase the difficulty of penetration.

A case study on expert systems in intrusion detection was presented by Michael Sebring, Eric Shellhouse, and Mary Hanna of NCSC and R. Whitehurst of SRI International. The Multics Intrusion Detection and Alerting System (MIDAS) is the expert system which provides real-time intrusion and misuse detection for NCSC’s networked mainframe Dockmaster. Using statistical profiles that characterize normal system and user behavior, one can detect system or user activity that deviates beyond certain limits. MIDAS employs this basic concept to evaluate the audited activities of more than 1200 Dockmaster users. W. Sibert of Oxford Systems presented the next paper which described the significant features of the SunOS MLS auditing mechanism and how it performs useful audit functions in large distributed systems. Although distributed systems pose difficulties in storing audit messages, Sibert concluded that the use of multiple buffers and failure recovery algorithms makes auditing practical and efficient in a distributed system.

### 2.4 Applying Security Techniques

Security issues assume greater significance as distributed systems of increasing size and complexity are built. Integrating security in a large distributed system was the subject of a talk by M. Satyanarayanan of Carnegie Mellon University. “Andrew” is a distributed computing environment which eventually will encompass over 5000 workstations at that university; it addresses issues such as the many levels of abstraction spanned, the need for compatibility, and the many detailed aspects of the system that are affected. Andrew offers substantially greater security than existing distributed systems without significant loss of usability or performance.

Ann Marmor-Squires and Patricia Rougeau of TRW Federal Systems Group presented a joint paper on process models and integrated environments for trusted system development for Defense needs. They concluded that several contributing technologies, when merged, achieve a better result than components alone could produce, such as a new risk-driven process model coupled with formal methods. John McDermott of the Naval Research Laboratory offered a technique for removing a class of Trojan horses from high-order languages. This defensive technique exploits the symbiotic relationship between the source text of the legitimate compiler and the self-reproducing feature of the Trojan horse object code. The technique is appropriate for very high assurance systems where every available defensive measure is desired.

In the area of communications security, Paul Lambert of Motorola described an architectural model of the Secure Data Network System (SDNS) Key Management Protocol. The SDNS project has developed a security architecture within the Organization of International Standardization’s (ISO) Open System Interconnection (OSI) computer network model. Within this architecture, the Key Management Protocol (KMP) provides a uniform mechanism for the establishment of secure communications. Lambert emphasized the security services furnished by the KMP and its relationship to the OSI reference model.

### 2.5 Verification

Crow, Lee, Rushby, von Henke, and Whitehurst of SRI International presented an over view of the Enhanced Hierarchical Development Methodology (EHDM) specification and verification system, a state-of-the-art environment designed specifically to meet the needs of security verification. Under development at SRI since 1983, EHDM incorporates many modern ideas and techniques concerning language design, specifications, and development environments in order to provide a state-of-the-art verification system. EHDM is currently used primarily in the SeaView project which combines element-level labeling with Al assurance. Other papers in this area concerned the State Delta Verification System (SDVS) and code level verification.

### 2.6 Database Management Security

Unisys Defense Systems researchers James Gray and James O’Connor have developed a distributed architecture for a multilevel secure database management system. The architecture is notable in two respects: its ability to include as a factor the effect of the security class of the query in the classification of derived data, allowing tuple level labels to be safely used for mandatory access control; and its provision of reliable tuple level labeling without requiring the relational operators to be trusted. This architecture thus becomes a suitable basis for a near-term solution to multilevel database management using existing DBMS components to implement the relational operators.

### 2.7 Networking and Local Area Networks

As more computer systems are interconnected through networks, the need for network security standards grows. NCSL’s Dennis Branstad chaired the networking session. Typical of this category of presentations was MITRE’s Kimberly Kirk-patrick’s talk on network security standards. In response to the need to provide security for each participant in an open system, various standards bodies are developing security-related standards within the context of the International Standards Organization/Open Systems Interconnection (ISO/OSI) reference model. Ms. Kirkpatrick summarized the security activities of the various standards groups as of May 1988, citing the structure, interactions, and work in process within each organization. She concluded that standards for security based on the ISO/OSI reference model are a fledgling area, with much work to be done before security standards reach the International Standard status.

Local area networks (LANs) are being widely used for a large number of applications. To address the vulnerability of networks such as an Ethernet to a variety of security threats, B. Herbison, Digital Equipment Corporation, reported on the Ethernet Enhanced-Security System. The system consists of Digital Ethernet Secure Network Controllers which are encryption devices and VAX Key Distribution software, which manages the controllers on an Ethernet or extended Ethernet and enforces a LAN access control policy. This system is effective in protecting against masquerading, wiretapping, and modification attacks, and, to a limited extent, some denial of service attacks.

### 2.8 System Security Requirements

Representative of this series of papers is one describing a secure software library for the Strategic Defense System (SDS), authored by Hadley, Hellwig, Rowe, and Vaurio of NCSC. Their presentation addressed the use and distribution of reusable software, a major challenge facing the Strategic Defense Initiative (SDI). The focus of their talk was the need and method of achieving a secure SDS software library where reusable software can be cataloged, accessed, and distributed. Other papers in this series dealt with the trusted military message processor, sensitivity labels and security profiles, and lessons learned in managing the accreditation process.

### 2.9 Automated Tools

Irene Gilbert of NCSL served as the session chair for automated tools. NCSL’s D. Richard Kuhn presented a paper describing a suite of static analysis tools for security certification. The tools are currently being used on software for secure Electronic Funds Transfer (EFT) but could be applied to other applications. NCSL has been assisting the Department of the Treasury by developing source code analysis tools to assist in the evaluation of software used in EFT equipment. The experimental tools described in this paper are being used on relatively small Treasury systems, but the research shows promise for expanding the use of tools for application to large systems.

### 2.10 Security Architectures

System security is considered stronger if based upon a hardware architecture that enforces Trusted Computing Base (TCB) constraints. Unisys Corporation’s Larry Ketcham described how a software-based security architecture can protect itself against programs that attempt to compromise system security. The focus is on issues involving the creation and protection of program code and the extent to which compilers are included in the TCB. Ketcham concluded that when the operating system, compilers, and hardware together are able to protect the integrity of the TCB, a multi-domain security architecture is achieved.

## 3. Conference Summary

“Computer Security … Into the Future” covered a range of broad issues and specific advanced technologies which enhance the integrity and security of automated information in government and industry today. The importance of planning for the security of tomorrow’s computer systems is critical to all who understand the value of their organization’s information resources.

Conference proceedings are available from conference co-chair Irene Gilbert, NCSL, A216 Technology Bldg., National Institute of Standards and Technology, Gaithersburg, MD 20899, or you may call (301) 975-3360.

